# Protective Role of Rheumatic Diseases Against Hepatitis B Virus Infection and Human Leukocyte Antigen B27 Highlighted

**DOI:** 10.3389/fmed.2022.814423

**Published:** 2022-02-10

**Authors:** Junna Ye, Peilin Xie, Zhuochao Zhou, Yue Sun, Fan Wang, Yijun You, Jialin Teng, Chengde Yang, Xinxin Zhang, Yue Han

**Affiliations:** ^1^Department of Rheumatology and Immunology, Ruijin Hospital, Shanghai Jiao Tong University School of Medicine, Shanghai, China; ^2^Department of Infectious Diseases, Research Laboratory of Clinical Virology, Ruijin Hospital, Shanghai Jiao Tong University School of Medicine, Shanghai, China; ^3^Sino-French Research Centre for Life Sciences and Genomics, Ruijin Hospital, Shanghai Jiao Tong University School of Medicine, Shanghai, China; ^4^Clinical Research Center, Ruijin Hospital, Shanghai Jiao Tong University School of Medicine, Shanghai, China

**Keywords:** rheumatic diseases, HBcAb, HLA-B27, ankylosing spondylitis, propensity score matching

## Abstract

**Background:**

By determining the hepatitis B virus (HBV) surface antigen (HBsAg) positive rate postexposure and HBV-specific antigen/antibody (Ag/Ab) level in patients with rheumatic diseases, we aimed at exploring the rheumatic link to HBV control.

**Methods:**

Patients who underwent HBV screening in the Ruijin Hospital from 2020 to 2021 were enrolled for the exposure rate estimation. Among antibody to HBV core antigen (HBcAb)-positive patients, we adopted propensity score matching (PSM) to study the impact of rheumatism on HBsAg seroprevalence after exposure. A second PSM evaluated the Ag/Ab differences. We also had HBsAg prevalence in human leukocyte antigen B2 (HLA-B27) tested patients studied.

**Results:**

With 33,989 screened patients, exposure rates remained comparable between rheumatic and non-rheumatic patients: 48.94 vs. 49.86%. PSM first yielded 2,618 balanced pairs. We observed significantly fewer patients with rheumatic diseases in HBsAg positive cases than negative ones (*p* < 0.001). In the second round, PSM matched 279 pairs, HBsAg (*p* < 0.001) and HBeAg (*p* < 0.05) positivity rates were significantly lower in the rheumatic patients, whereas HBsAb positivity rate (*p* < 0.001) and level (*p* < 0.01) were significantly higher. Though the value of HBcAb was overall significantly lower (*p* < 0.001) within the realm of rheumatic diseases, patients with ankylosing spondylitis (AS) demonstrated a significantly higher value than other rheumatic diseases. We saw significantly fewer HBV infections in HLA-B27 positive subjects than in the negative ones (*p* < 0.001).

**Conclusion:**

In this propensity score-matched study, rheumatic patients had an advantage in HBV control. In rheumatic patients, HBcAb levels, together with the beneficial role of HLA-B27, were highlighted.

## Introduction

The WHO estimates 296 million people had chronic hepatitis B infection in 2019. Hepatitis B virus (HBV) can cause persistent infection, ultimately leading to cirrhosis and hepatocellular carcinoma (1). Currently, there is no sterilizing cure. HBV, by evolving host mimicking strategies, causes inertia in host immunity. Thus, it is the possibility of a systemic rescue and it has been indeed evidenced by the fact that CD8+ T cells dysfunction induced locally by hepatocellular priming can be counteracted *in vivo* by treatment with IL-2 (2). CD8+ T cells are considered the ultimate effectors of viral clearance.

While HBV leads to immune tolerance, hyperactive immunity or breakage of self-tolerance is the centerpiece of rheumatic diseases, often associated with the perturbated humoral immunity (3). The impact of rheumatic disease on HBV infection outcome had never been studied, nor had a rheumatic viral-specific humoral profile. Thus, one is curious about the product of having both conditions. Whether this overactivated immunity could compensate for the deficiency seen in non-rheumatic patients and lead to better control over HBV remains unclear. Indeed, shreds of evidence had suggested that the reactivation rate of HBV in patients with rheumatic diseases under immunosuppressive agents was low (4). If any, the prognostics were overall good, compared with non-rheumatic patients (5). However, data regarding the actual prevalence of HBsAg seropositivity after encountering HBV among rheumatic patients are scarce (6). Neither had any study evaluated impact of rheumatism on HBsAg seroprevalence in HBV exposed population, nor had any of them cross-sectionally compared either the exposure or the infection rate between rheumatic and non-rheumatic groups. Thus, the consequence is unclear.

The input of humoral immunity has drawn attention recently (7) in the field of HBV. HBV virion is an enveloped nucleocapsid. HBV surface antigen (HBsAg) forms the envelope. Antibody to HBsAg, HBsAb, is an indicator of protective immunity. It appears after viral clearance in animal models (8). Conversely, the antibody against nucleocapsid forming core protein antigen-HBcAg, HBcAb, was produced early in infection (9). The significance and kinetics of HBcAb, and other antibodies against core coding products, including Hepatitis B e antigen (HBeAg), is unclear (10). We previously found that overactivated HBV immunity in patients could be HBV core coding proteins related (11). We also discovered that the HBcAb level possessed a prognostic value in severe cases (12), albeit in a biphasic manner.

Here, by applying propensity score matching (PSM) on a large dataset of HBV serologically screened population, we tested the hypothesis that after encountering HBV, rheumatic patients showed less HBsAg prevalence compared with non-rheumatic patients. We compared HBV-specific antigen/antibody (Ag/Ab) to search for rheumatic traits against HBV. The protective role of human leukocyte antigen B27 (HLA-B27) was further validated.

## Materials and Methods

### Study Populations

**Figure 1:**
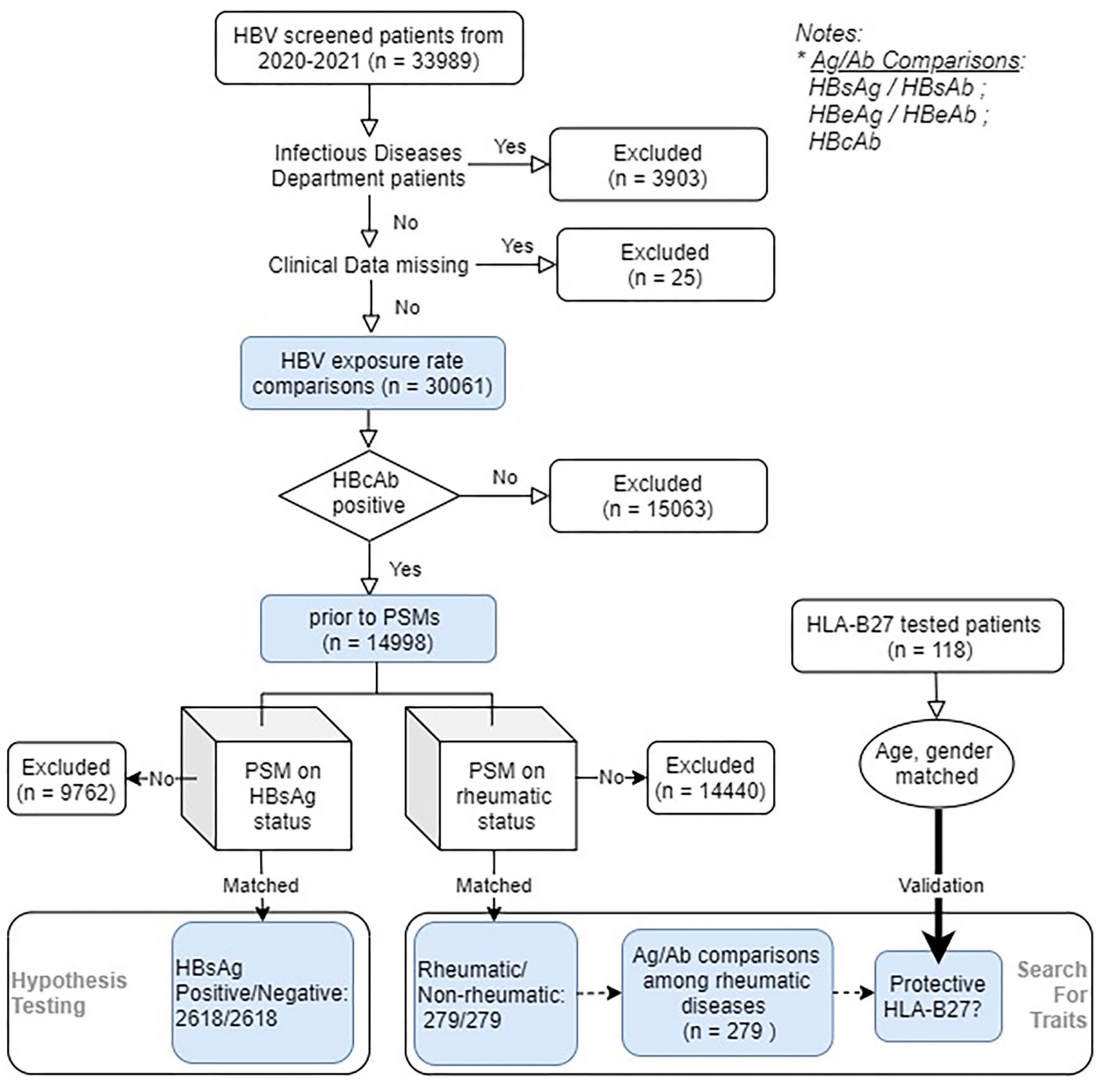
Flowchart of the study. A total of 33,989 eligible hepatitis B Virus (HBV) serologically screened patients entered the study (rheumatic/non-rheumatic patients: 567/33,422), within which 17,906 patients were HBV core antigen (HBcAb) positive. Initially included patients were used to estimate the HBV exposure rates. Among HBcAb-positive patients (HBV-exposed patients), two parallel rounds of PSM were conducted. The first one aimed to check the impact of rheumatism on the infection outcome, whereas the second one focused on evaluating the HBV-specific humoral immunity [viral antigen (Ag)/antibody (Ab)] differences. Viral Ab levels were further compared among different rheumatic diseases. The role of human leukocyte antigen (HLA) class I molecule B27 (HLA-B27) on HBV infection outcome was further validated using another set of age, gender-matched patients.

We included 33,989 HBV serological markers screened patients from March 2020 to March 2021 in the Ruijin Hospital, Shanghai, China. Figure 1 demonstrates the study outline. The validation group consisted of patients who had HLA-B27 screened. All the rheumatic disease diagnoses were captured by rheumatologists. Each of them was then confirmed by two experienced rheumatologists to ensure the correctness of classification. Patients with vague or missing data as well as those with more than one rheumatic diagnosis were excluded. Ankylosing spondylitis (AS) was diagnosed according to the Assessment of SpondyloArthritis International Society/European League Against Rheumatism (ASAS-EULAR) recommendations (13), systemic lupus erythematosus (SLE) according to 2019 European Alliance of Associations for Rheumatology/American College of Rheumatology (EULAR/ACR) classification criteria (14), RA based on 2010 ACR/EULAR classification criteria (15), Sjögren's syndrome (SS) based on 2016 ACR/EULAR criteria (16), dermatomyositis (DM) by using the classification criteria of 2017 EULAR/ACR (17), and vasculitis according to ACR/EULAR-endorsed diagnostic and classification criteria (18). HBV reactivation was defined according to the 2020 BMJ review (19). The study was approved by the Ethics Committee of Ruijin Hospital. Medical charts of all the matched rheumatic cases were carefully scrutinized, treatment options, and reactivation were recorded.

### Hepatitis B Virus Serological Markers and HLA-B27 Detection

Hepatitis B virus surface antigen, HBsAb, HBeAg, HBeAb, and HBcAb were measured by chemiluminescence using the Architect i2000SR Immunoassay Analyzer (Abbott Diagnostics, Abbott Park, Illinois, USA). Results of HBsAg were presented in IU/ml and HBsAb in mIU/ml, both with corresponding ranges. The results of HBeAg, HBeAb, and HBcAb were all presented in relative luminescence unit signal over cutoff ratio (s/co). Cutoff value and ranges were as follows: 0.05 and 0.0–250 IU/ml for HBsAg; 10 and 0.00–1,000.00 mIU/ml for HBsAb; 1.00 s/co as the cutoff for HBeAg and HBcAb, a value higher than 1.00 was considered as positive; 1.00 s/co set as the cutoff for HBeAb, a value higher than 1.00 were defined as negative. If a result fell in the gray zone, the sample was subjected to duplicate retesting and considered repeatedly reactive when both turned reactive. If both the retests were negative, we considered the original sample negative. When contradictory results appeared, we initiated external validation by Cobas e 801 analytical unit for immunoassay tests (Roche Diagnostics, Indianapolis, USA). HLA-B27 positivity was determined in the blood samples by the flow cytometric approach.

### Sample Size Evaluation

To compare the exposure outcome, samples size was computed using *pwr.chisq.test* function from *pwr*, an *R* package, with significance level/power set to 0.05/0.8. According to preliminary bootstrapped calculations, effect size was constantly >0.5. Still, a medium effect size value of 0.2 was chosen, yielding 273 as the minimal required number of observations. For visualization, Supplementary Figure 1 plots the power calculation.

### Statistical Analysis—Propensity Score Matching

To obtain less biased inferences, we used PSM (Rosenbaum and Rubin, 1983) to estimate the effect of rheumatism on HBV infection outcome (HBsAg status as binary outcome) on those who had been exposed to HBV for confounding by the included covariates. The selection of covariates followed the principle that it should be related to the outcome and unrelated to the exposure. Age is a determinant of serologic responses to virus infection, and so is gender. Standardized mean differences, variance ratios, and empirical cumulative density function were checked on these two covariates using the initial dataset. Both age and gender were significantly different between the outcome groups. Being younger or male was prone to infection. Therefore, they needed to be balanced before any inference could be made. PSM can reduce covariates to one dimension. We attempted 1:1 nearest neighbor PSM without replacement with a propensity score estimated using logistic regression, which yielded adequate balance, as visualized in Supplementary Figure 2 by quantile–quantile plots and Supplementary Figure 3 by the distribution of propensity scores of matched and unmatched units. After matching, to estimate the effect and its standard error, we fit a linear regression model with infection outcome and rheumatism and the covariates as additive predictors. The *lm()* function was used to estimate the effect. The second PSM was performed to balance the rheumatic and non-rheumatic groups to study the viral Ag/Ab difference on having rheumatic diseases. After this matching, groups were also balance-checked by graphic means. Exact matching was performed using the *MatchIt* package (20) in *R*. The Pearson's Chi-Squared test with a Yates' continuity correction was used to test the goodnessof-fit whether a categorical variable followed a hypothesized distribution. All the statistics were performed in *Rstudio* software (version 1.4.1106, Massachusetts, USA).

### Influence of Rheumatic Diseases on HBV Infection Outcome and Comparison of HBV Antibody Levels

To assess the influence of rheumatism on HBsAg prevalence, we applied the chi-squared test on the first PSM-yielded covariates balanced case-control pairs. Comparisons of positivity rates of HBV serological markers between patients with or without rheumatic diseases were performed on the second PSM balanced pairs. The patients were further divided into the female and male subgroups and HBV positivity rates of the serological markers were compared. The levels of HBsAb, HBeAb, and HBcAb were also analyzed. With regards to the differences between antibodies levels (HBsAb, HBeAb, and HBcAb), after normality check, they were subjected to inter- or intra-disciplinary comparisons. Non-parametric data were compared using the Welch *t-*test. All the tests were two-sided, with a significance level <0.05.

### Comparison of HLA-B27 Status on HBV Infection Outcome

To test whether HLA-B27 status influenced infection outcome, HBsAg/HBeAg/HBV DNA status, and also HBsAb/HBeAb/HBcAb levels were compared between HLA-B27 positive and negative groups. Comparability of gender and age between the two groups was chi-squared or *t*-tested first. In the downstream analysis, for HBsAg, HBeAg, or detectable HBV DNA, the chi-squared test was applied, for HBsAb level, HBcAb s/co, and HBeAb s/co value, *t*-test was used. All the tests were two-sided, with a significance level <0.05.

## Results

### Comparable HBV Exposure Rates Among Different Groups of Patients

**Figure 2:**
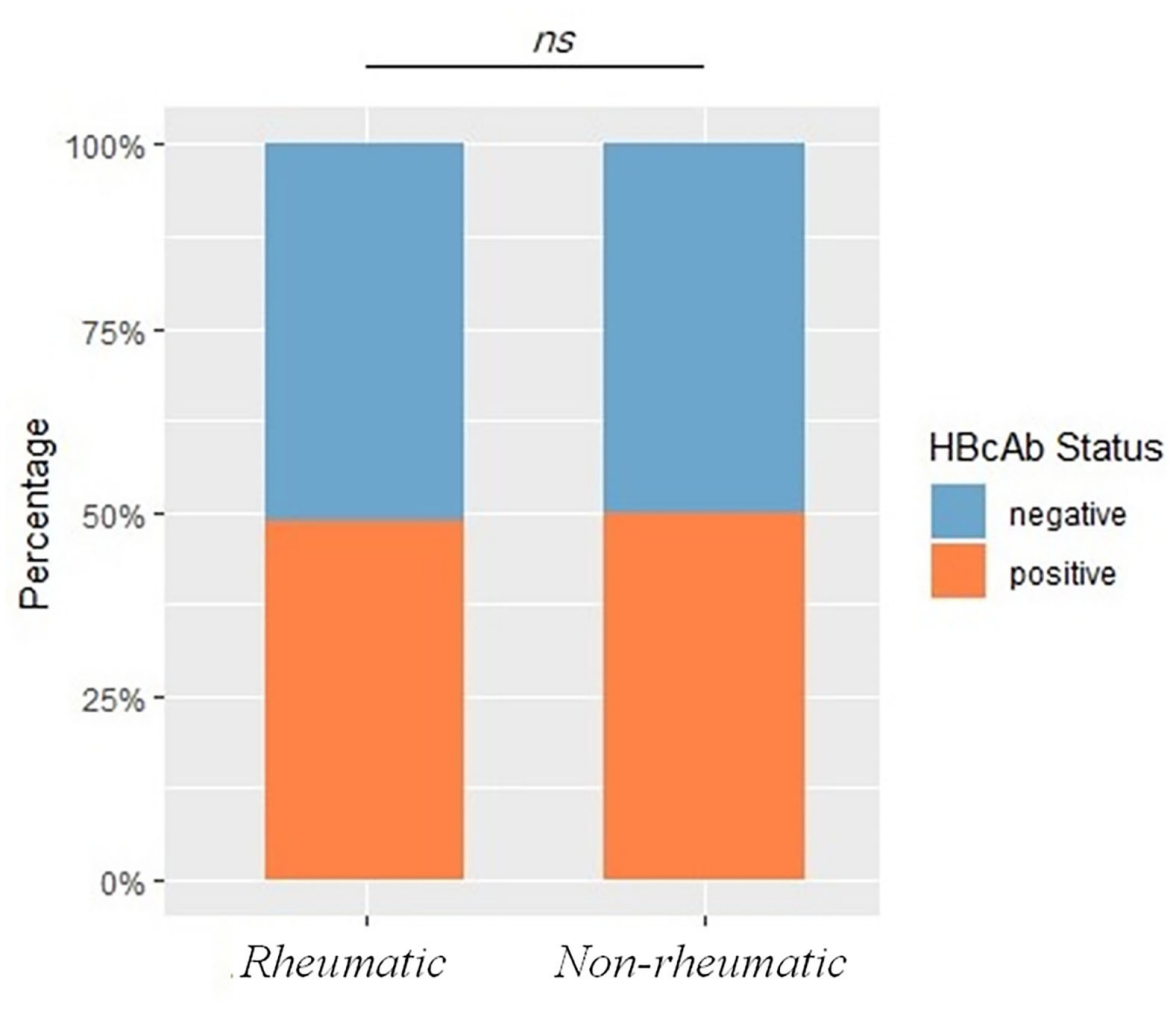
Exposure rates between rheumatic and non-rheumatic patients and demographics before propensity score matching (PSM). The bar plot shows HBV exposure rates, represented by HBcAb status, between rheumatic and non-rheumatic patients. A *p* < 0.05 was considered as statistically significant. Ns stood for non-significance.

Hepatitis B virus core antigen HBcAb positivity rate was 48.94% in patients with rheumatic diseases, comparable with 52.74% in non-rheumatic departments (including patients from the Department of Infectious Diseases, *p* = 0.07), within which patients from the Department of Infectious Diseases had a significantly enriched HBcAb positive rate of 74.51% (*p* < 0.0001). Exposure rates did stay comparable (see later) with or without patients from the Department of Infectious Diseases, a possible consequence of dilution of the large sample size. Because patients with a previously established diagnosis of HBV infection would be referred to the Infectious Diseases Department or seeking further consultations by themselves, thus including these patients would inevitably introduce and propagate inclusion bias. These subjects were excluded from the non-rheumatic group for further analysis. HBcAb positivity rate stayed comparable between rheumatic and non-rheumatic patients (48.94 vs. 49.86%, *p* = 0.66), as shown in Figure 2, demographics and Ag/Ab comparisons before PSM were listed in Table 1.

**Table 1:** Demographic comparison between rheumatic and non-rheumatic patients prior to matching.

	**Rheumatic patients**	**Non-Rheumatic patients**	***p-value***
**Exposure rate comparison stage**			
Age (years old, mean)	48.14	51.93	<0.001
Gender (Female/Male)	363/205	16,513/12,980	<0.001
HBsAg status (Positive/Negative)	20/548	2,615/26,878	<0.001
HBcAb status (Positive/Negative)	279/289	14,719/14,774	0.742
Prior to PSM^*^			
Age (years old, mean)	53.86	57.58	<0.001
Gender (Female/Male)	177/102	7,348/7,371	<0.001
HBsAg status (Positive/Negative)	19/260	2,599/12,120	<0.001

* *Age and gender were matched after propensity score matching (PSM)*.

### Patients With Rheumatic Diseases Less Seen in HBsAg-Positive HBV Exposed Subjects

**Figure 3:**
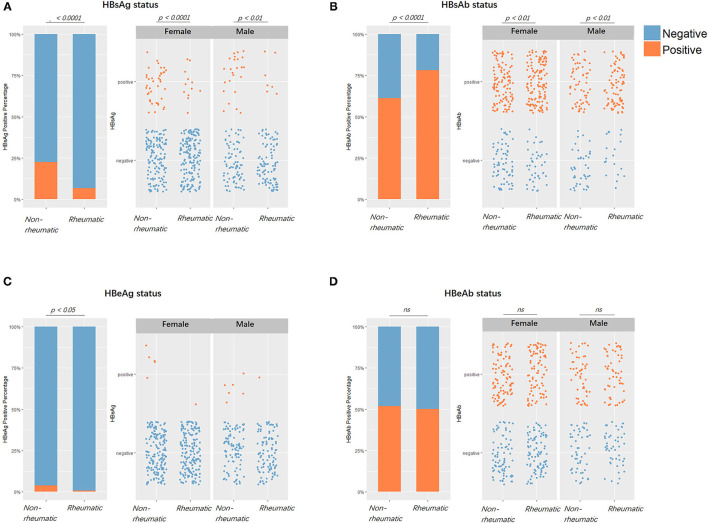
Viral markers positivity rates comparisons between patients with rheumatic and non-rheumatic diseases. **(A)** From left to right, the first figure showed HBV surface antigen (HBsAg) positive/negative status percentages between the rheumatic and non-rheumatic groups. Blue stood for negative serological results, while orange stood for positive results. The second and third figures showed subgroup results of females and males. Each point represented one patient. From **(B–D)**, the positive/negative status of HBsAb, HBeAg, and HBeAb in patients from the two groups along with gender subgroups were laid out in the same sequence as in **(A)**.

The PSM on HBsAg status yielded 2,618 balanced case-control pairs. Between the HBsAg-positive and negative groups, rheumatic patients were significantly less seen in the positive group (*X*^2^ = 214.84, *df* = 1*, p* < 0.0001). The PSM on rheumatic status provided 279 balanced pairs. Noteworthy, the HBsAg positivity rate stayed significantly lower in the rheumatic group compared with the non-rheumatic group regardless of matching. Before matching was 6.81 vs. 17.7% (*p* < 0.0001) and after matching was 6.81 vs. 22.58% (*p* < 0.0001). HBsAb positivity was significantly more frequently (*p* < 0.0001) seen in the rheumatic group than in the non-rheumatic group. HBeAg positive cases were less seen (*p* < 0.0001) in the rheumatic (HBeAg positive/negative: 2/277) than in the non-rheumatic group (11/268). However, there was no significant difference in terms of HBeAb positivity rate (*p* = 0.73; Figure 3).

### Rheumatic Patients Produced a Higher Level of HBsAb and a Lower Level of HBcAb

**Figure 4:**
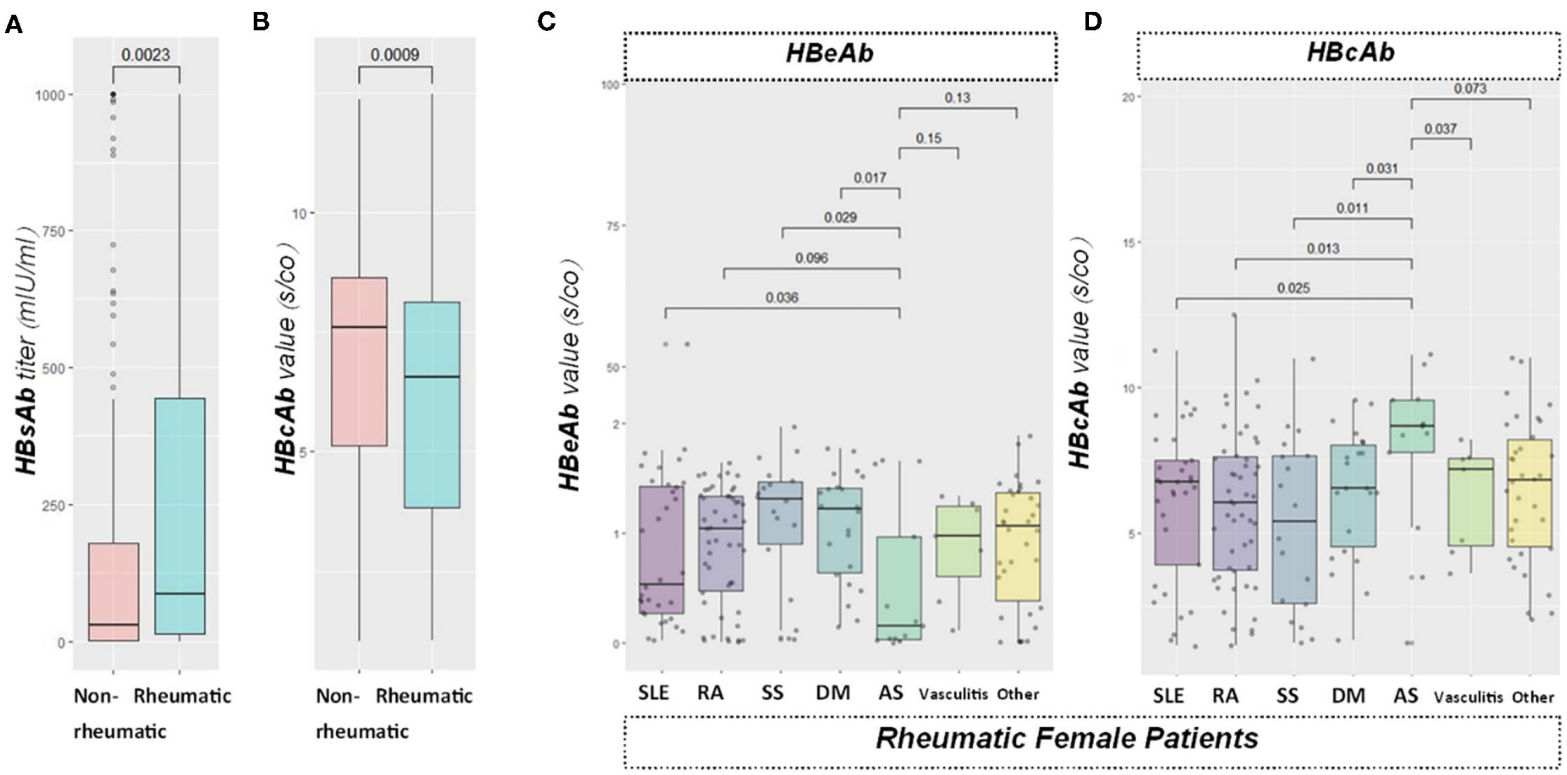
Viral specific antibody level comparisons. From left to right: **(A)** boxplot showed HBsAb value between the rheumatic and non-rheumatic groups. **(B)** boxplot showed HBcAb signal over cutoff ratio (s/co) value in patients from the two groups; **(C,D)** boxplots sequentially showed HBeAb and HBcAb antibodies levels among different rheumatic diseases. HBeAb values were significantly different between ankylosing spondylitis (AS) with systemic lupus erythematosus (SLE), Sjögren's syndrome (SS), or dermatomyositis (DM) group in the female subgroup, while no significance was found in the male group. Moreover, a significantly higher value of HBcAb in patients with AS than SLE, RA, SS, DM, or vasculitis in the female subgroup were also found, while no significance was found in the male group.

Our data demonstrated that the HBsAb level was significantly higher (*p* < 0.01) in the rheumatic group than in the non-rheumatic group. The difference remained in the gender subset (*p* < 0.05 in females, 0.01 in males). HBeAb s/co value was comparable (*p* = 0.37) in these two groups. Gender subgroups did not reveal any further significant difference (*p* = 0.30 in female subgroup, *p* = 0.89 in male subgroup). HBcAb s/co value was found to be significantly lower (*p* < 0.0001) in the rheumatic (6.06 s/co) than in the non-rheumatic group (6.82 s/co). Moreover, there was a significant difference in the female subgroup (*p* < 0.01 in females, *p* = 0.05 in males) (Figure 4).

### Higher HBcAb Level in Female Ankylosing Spondylitis (AS) Patients Than That in Other Rheumatic Diseases

Rheumatic disorders included in this study encompassed SLE, RA, SS, DM, AS, vasculitis, and others. A total of 212/279 (75.99%) of rheumatic patients were already under immunosuppressants. We found no record of HBV reactivation or use of preemptive antivirals. No significant difference was found with pairwise comparisons between different rheumatic diseases regarding HBsAb levels. Significant differences were noticed in the female subgroup when the HBeAb s/co value was compared between AS and SLE (*p* < 0.05), SS (*p* < 0.05), or DM (*p* < 0.05). Female AS subjects had significantly lower HBeAb s/co value (inversely related to the actual antibody level) than that in SLE/SS/DM groups. Nevertheless, no significant difference was found in the male subset. In addition, patients with AS had higher HBcAb s/co value than patients with SLE (*p* < 0.05, RA (*p* < 0.05), SS (*p* < 0.051), DM (*p* < 0.05) or vasculitis (*p* < 0.05; Figure 4). However, we found no significant difference in males.

### HLA-B27 Positive Subjects Had Better Control Over HBV Infection

**Figure 5:**
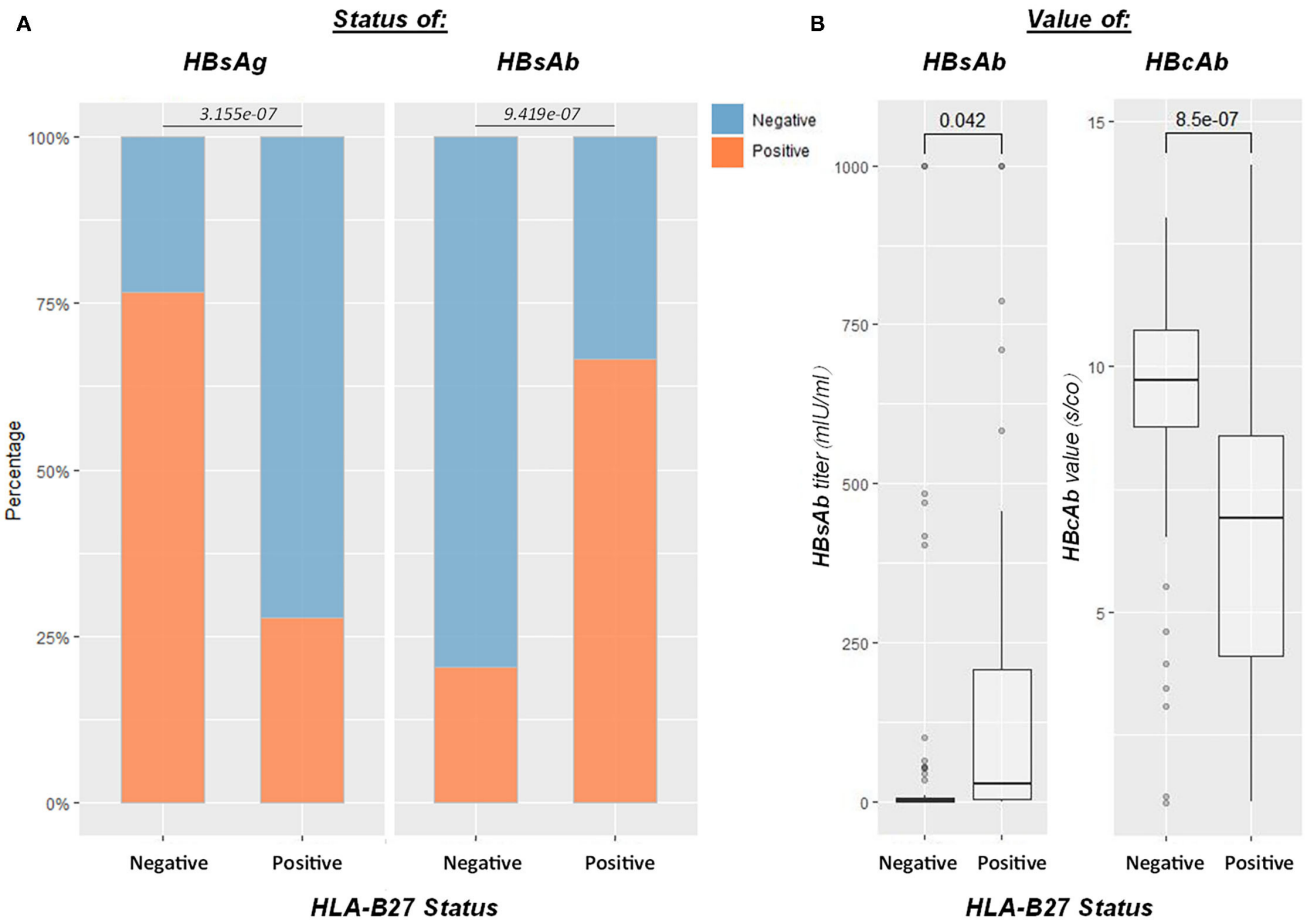
Influence of HLA-B27 positivity on the HBV infection outcome. Efficient HBV control in HLA-B27-positive patients (*n* = 118 for HBcAb-positive patients who underwent HLA-B27 screen). **(A)** From left to right, plots showed HBsAg, HBsAb status between HLA-B27 positive and negative patients, respectively; **(B)** the last two boxplots demonstrated HBsAb and HBcAb levels between HLA-B27 negative and positive patients. HLA-B27 positive patients had significantly higher HBsAb and lower HBcAb than negative ones.

Human leukocyte antigen B27 was a biomarker for AS. To further analyze the findings on AS, we enrolled 201 HLA-B27 and HBV-tested patients. A total of 118 patients had positive HBcAb, within which 54 patients were HLA-B27 positive, 64 were negative. Age (*p* = 0.97) and gender (*p* = 0.67) were comparable between the two groups. HBsAg positivity rate was significantly lower in the HLA-B27 positive group (*p* < 0.0001), and the average HBsAb level *(p* = 0.04) was notably higher than that of HLA-B27 negative patients. HBeAg positivity rates were comparable between HLA-B27 negative and positive patients (*p* = 0.60). However, HBeAb positivity rates in HLA-B27 positive patients were significantly lower than HLA-B27 negative patients (*p* < 0.01). Besides, we encountered less detectable HBV DNA in HLA-B27 positive patients (*p* < 0.0001). HBcAb s/co value was significantly lower (*p* < 0.0001) in the HLA-B27 positive group than in the HLA-B27 negative group. No difference was found in HBeAb s/co value in these two groups (*p* = 0.56; Figure 5). None of the HLA-B27 positive patients developed a persistent infection after HBV exposure.

## Discussion

Our data suggested a beneficial role of rheumatic diseases on HBV control. We further discovered a protective input of HLA-B27. The immunopathogenic determinants governing HBV infection outcome remain poorly understood. A hallmark of rheumatic diseases is unrestrained autoimmunity, often associated with the overproduction of autoantibodies (21), especially in females (22). These groups of patients provide us the opportunity to evaluate a derailed immunity's impact on infection outcome. Since a study on HBV-specific antibody levels in patients with rheumatic diseases (23) was unavailable, we evaluated their differences, in the rheumatic vs. non-rheumatic groups. Intriguingly, two phenomena are worth noticing. First, the significantly higher HBsAb level evidenced in rheumatic patients, indicative of more successful control, contradicted the fact that HBcAb was exceptionally lower. Second, though relatively lower, levels of HBcAb in patients with AS were higher compared with other rheumatic diseases, especially in females. Current knowledge of antibodies against core-coding proteins, including HBcAb, during the course of infection, is limited. The previous study had shown that HBV-exposed patients with AS reactivated even less compared with other rheumatic conditions (24), suggesting superior control over HBV. Since HLA-B27 positivity is unequivocally a cardinal sign of AS, the unusual HBcAb levels in patients with AS imply a possible link between this HLA-class I molecule and HBV infection. Following these leads, we further tested the protectiveness of HLA-B27, and it was validated subsequently.

Human cells express diverse HLA class I molecules to present CD8^+^ T cell epitopes. Should there be an interaction between HBV core antigen coding proteins and HLA-class I molecule, it would be during the T cell priming by cross-presentation of the viral-specific peptide. Cross-presentation requires an HLA-class I molecule. In the case of HBV infection, this can be achieved either by antigen-presenting cells or non-professional cells such as hepatocytes. This leads to intrahepatic priming of the effector CD8^+^ T cell. It is crucial to viral control. Polymorphism of HLA affects antigen recognition. Its potential role in defining HBV infection outcome had been suggested, except mostly covered were HLA class II loci (25). Still, a plausible link between HLA-B and HBV had been recognized by a few studies (26). One of them, by probing into HBV core escape mutants and HLA class I alleles, found a handful of sites within the HBV PreC/C gene under selection pressure derived from HLA-B (27). Another recent study found HLA-class I profile heavily influenced HBV-specific T cell composition (28). For now, we are aware that HBsAg and HBeAg can be tolerogenic. However, we know little regarding which part of HBV virion can trigger an effective adaptive immunity. HLA-B27 (subtypes B^*^2701-2759) belongs to class I surface antigen encoded by the B locus in the major histocompatibility complex on chromosome 6 and presents antigenic peptides (self/non–self-antigens) to T cells. Though tightly associated, its relationship with AS has not been fully elucidated. Notably, HLA-B27 is strongly enriched among HIV controllers (29), suggesting its beneficial role in the fight against HIV, which is also a reverse transcribed virus, like HBV. Being on the humoral branch of adaptive immunity, antibody to the core coding antigen, HBcAb, some proposed that it was related to inflammation (30), others found its baseline level predicted reactivation under immunotherapy (31). We previously demonstrated the role of core coding protein in immunopathogenesis (3). We also found that severe cases of HBV infection had higher HBcAb levels, while in the terminal stage, lower HBcAb level was correlated with poor outcomes (12). Based on these considerations, the immunogenicity of core-related proteins during HBV infection might be complex, yet important.

Indeed, there are other study design options with their strengths and limitation. Those ideal ones can be cumbersome. We had adopted PSM on this cross-sectional study to minimize the bias. Still, whether HLA-B along with core coding proteins could be the hinge to an effective HBV surveillance and elimination needs to be addressed with a future experimental study. A better understanding of these could help to identify factors at work in HBV pathogenesis and lead to new treatments.

Taken together, rheumatic patients shared advantage in the control over HBV, given the same exposure rate. Lower antibodies levels against viral core gene coding proteins in the rheumatic patients with the protective role of HLA-B27 shed light in the future experimental design to unearth the possible signaling interaction between the HLA-B allele and HbcAg.

## Data Availability Statement

The original contributions presented in the study are included in the article/Supplementary Material, further inquiries can be directed to the corresponding author/s.

## Ethics Statement

The studies involving human participants were reviewed and approved by the Ethics Committee of Shanghai Ruijin Hospital. The patients/participants provided their written informed consent to participate in this study.

## Author Contributions

YH and JY: conceived the study. YH, PX, YS, FW, YY, and JT: extracted the data. YH: devised the methodology and did the formal analysis, wrote the original draft of the manuscript, and reviewed and edited the manuscript. YH, CY, XZ, and JY: acquired funding. CY, XZ, and YH: supervised the study. ZZ: prepared the figures. All authors contributed to the final version of the manuscript and approved it for publication.

## Funding

This study has been funded by National Natural Science Foundation of China (82002126, 81974301, 81801592, and 82101876), the Clinical Research Plan of SHDC (SHDC2020CR4011), the Ruijin Hospital Youth Incubation Project (KY2021607), and the Shanghai Pujiang Young Rheumatologists Training Program (SPROG202006).

## Conflict of Interest

The authors declare that the research was conducted in the absence of any commercial or financial relationships that could be construed as a potential conflict of interest.

## Publisher's Note

All claims expressed in this article are solely those of the authors and do not necessarily represent those of their affiliated organizations, or those of the publisher, the editors and the reviewers. Any product that may be evaluated in this article, or claim that may be made by its manufacturer, is not guaranteed or endorsed by the publisher.

## References

[1] Iannacone M Guidotti LG. Immunobiology and pathogenesis of hepatitis B virus infection. Nat Rev Immunol. (2021) 22:19–32. 10.1038/s41577-021-00549-4 34002067

[2] Benechet AP De Simone G Di Lucia P Cilenti F Barbiera G Le Bert N. Dynamics and genomic landscape of CD8(+) T cells undergoing hepatic priming. Nature. (2019) 574:200–5. 10.1038/s41586-019-1620-6 31582858 PMC6858885

[3] Apel F Zychlinsky A Kenny EF. The role of neutrophil extracellular traps in rheumatic diseases. Nat Rev Rheumatol. (2018) 14:467–75. 10.1038/s41584-018-0039-z 29930301

[4] Chen MH Chen MH Chou CT Hou MC Tsai CY Huang YH. Low but long-lasting risk of reversal of seroconversion in patients with rheumatoid arthritis receiving immunosuppressive therapy. Clin Gastroenterol Hepatol. (2020) 18:2573–81.e1. 10.1016/j.cgh.2020.03.039 32205219

[5] Yang C Xie M Zhang K Liu H Liang A Young KH. Risk of HBV reactivation post CD19-CAR-T cell therapy in DLBCL patients with concomitant chronic HBV infection. Leukemia. (2020) 34:3055–9. 10.1038/s41375-020-0913-y 32533094

[6] Ditto MC Parisi S Varisco V Talotta R Batticciotto A Antivalle M. Prevalence of hepatitis B virus infection and risk of reactivation in rheumatic population undergoing biological therapy. Clin Exp Rheumatol. (2021) 39:546–54. 32940216

[7] Burton AR Pallett LJ McCoy LE Suveizdyte K Amin OE Swadling L. Circulating and intrahepatic antiviral B cells are defective in hepatitis B. J Clin Invest. (2018) 128:4588–603. 10.1172/JCI121960 30091725 PMC6159997

[8] Asabe S Wieland SF Chattopadhyay PK Roederer M Engle RE Purcell RH. The size of the viral inoculum contributes to the outcome of hepatitis B virus infection. J Virol. (2009) 83:9652–62. 10.1128/JVI.00867-09 19625407 PMC2748002

[9] Maini MK Burton AR. Restoring, releasing or replacing adaptive immunity in chronic hepatitis B. Nat Rev Gastroenterol Hepatol. (2019) 16:662–75. 10.1038/s41575-019-0196-9 31548710

[10] Le Bert N Salimzadeh L Gill US Dutertre CA Facchetti F Tan A. Comparative characterization of B cells specific for HBV nucleocapsid and envelope proteins in patients with chronic hepatitis B. J Hepatol. (2020) 72:34–44. 10.1016/j.jhep.2019.07.015 31348999

[11] Xue Y Wang MJ Yang ZT Yu DM Han Y Huang D. Clinical features and viral quasispecies characteristics associated with infection by the hepatitis B virus G145R immune escape mutant. Emerg Micro Infect. (2017) 6:e15. 10.1038/emi.2017.2 28325923 PMC5378923

[12] Li J Gong QM Xie PL Lin JY Chen J Wei D. Prognostic value of anti-HBc quantification in hepatitis B virus related acute-on-chronic liver failure. J Gastroenterol Hepatol. (2021) 36:1291–9. 10.1111/jgh.15310 33091955

[13] van der Heijde D Ramiro S Landewe R Baraliakos X Van den Bosch F Sepriano A. 2016 update of the ASAS-EULAR management recommendations for axial spondyloarthritis. Ann Rheum Dis. (2017) 76:978–91. 10.1136/annrheumdis-2016-210770 28087505

[14] Aringer M Costenbader K Daikh D Brinks R Mosca M Ramsey-Goldman R. 2019 European league against rheumatism/American college of rheumatology classification criteria for systemic lupus erythematosus. Arthritis Rheumatol. (2019) 71:1400–12. 10.1002/art.40930 31385462 PMC6827566

[15] Aletaha D Neogi T Silman AJ Funovits J Felson DT Bingham CO III. 2010 rheumatoid arthritis classification criteria: an American College of rheumatology/European league against rheumatism collaborative initiative. Ann Rheum Dis. (2010) 69:1580–8. 10.1136/ard.2010.138461 20699241

[16] Shiboski CH Shiboski SC Seror R Criswell LA Labetoulle M Lietman TM. 2016 American college of rheumatology/European league against rheumatism classification criteria for primary sjogren's syndrome: a consensus and data-driven methodology involving three international patient cohorts. Arthritis Rheumatol. (2017) 69:35–45. 10.1002/art.39859 27785888 PMC5650478

[17] Lundberg IE Tjarnlund A Bottai M Werth VP Pilkington C de Visser M. 2017 European league against rheumatism/american college of rheumatology classification criteria for adult and juvenile idiopathic inflammatory myopathies and their major subgroups. Arthritis Rheumatol. (2017) 69:2271–82. 10.1002/art.40320 29106061 PMC5846474

[18] Craven A Robson J Ponte C Grayson PC Suppiah R Judge A. ACR/EULAR-endorsed study to develop diagnostic and classification criteria for vasculitis (DCVAS). Clin Exp Nephrol. (2013) 17:619–21. 10.1007/s10157-013-0854-0 23996327

[19] Shi Y Zheng M. Hepatitis B virus persistence and reactivation. BMJ. (2020) 370:m2200. 10.1136/bmj.m2200 32873599

[20] Ho D Imai K King G Stuart E. Matching as nonparametric preprocessing for reducing model dependence in parametric causal inference. Pol Anal. (2007) 15:199–236. Available online at: http://gking.harvard.edu/files/abs/matchp-abs.shtml

[21] Deng J Wei Y Fonseca VR Graca L Yu D. T follicular helper cells and T follicular regulatory cells in rheumatic diseases. Nat Rev Rheumatol. (2019) 15:475–90. 10.1038/s41584-019-0254-2 31289377

[22] Zhao R Chen X Ma W Zhang J Guo J Zhong X. A GPR174-CCL21 module imparts sexual dimorphism to humoral immunity. Nature. (2020) 577:416–20. 10.1038/s41586-019-1873-0 31875850

[23] Reddy KR Beavers KL Hammond SP Lim JK Falck-Ytter YT American Gastroenterological Association I. American gastroenterological association institute guideline on the prevention and treatment of hepatitis B virus reactivation during immunosuppressive drug therapy. Gastroenterology. (2015) 148:215–9; quiz.e16–7. 10.1053/j.gastro.2014.10.039 25447850

[24] Lee YH Bae SC Song GG. Hepatitis B virus (HBV) reactivation in rheumatic patients with hepatitis core antigen (HBV occult carriers) undergoing anti-tumor necrosis factor therapy. Clin Exp Rheumatol. (2013) 31:118–21. 23111095

[25] Hu Z Liu Y Zhai X Dai J Jin G Wang L. New loci associated with chronic hepatitis B virus infection in Han Chinese. Nat Genet. (2013) 45:1499–503. 10.1038/ng.2809 24162738

[26] Di Bona D Pandey JP Aiello A Bilancia M Candore G Caruso C. The immunoglobulin gamma marker 17 allotype and KIR/HLA genes prevent the development of chronic hepatitis B in humans. Immunology. (2020) 159:178–82. 10.1111/imm.13133 31613998 PMC6954734

[27] Abbott WG Tsai P Leung E Trevarton A Ofanoa M Hornell J. Associations between HLA class I alleles and escape mutations in the hepatitis B virus core gene in New Zealand-resident tongans. J Virol. (2010) 84:621–9. 10.1128/JVI.01471-09 19846510 PMC2798394

[28] Bertoletti A Ferrari C. Adaptive immunity in HBV infection. J Hepatol. (2016) 64 (1 Suppl):S71–83. 10.1016/j.jhep.2016.01.026 27084039

[29] Deeks SG Walker BD. Human immunodeficiency virus controllers: mechanisms of durable virus control in the absence of antiretroviral therapy. Immunity. (2007) 27:406–16. 10.1016/j.immuni.2007.08.010 17892849

[30] Song LW Liu PG Liu CJ Zhang TY Cheng XD Wu HL. Quantitative hepatitis B core antibody levels in the natural history of hepatitis B virus infection. Clin Microbiol Infect. (2015) 21:197–203. 10.1016/j.cmi.2014.10.002 25658546

[31] Yang HC Tsou HH Pei SN Chang CS Chen JH Yao M. Quantification of HBV core antibodies may help predict HBV reactivation in patients with lymphoma and resolved HBV infection. J Hepatol. (2018) 69:286–92. 10.1016/j.jhep.2018.02.033 29551710

