# Supplementary material for: Protective Role of Rheumatic Diseases Against Hepatitis B Virus Infection and Human Leukocyte Antigen B27 Highlighted
Source: Front Med (Lausanne). 2022 Feb 10;9:814423. doi: 10.3389/fmed.2022.814423 (PMC8867399; doi:10.3389/fmed.2022.814423)

**Supplementary figure 1: Sample size calculation:**


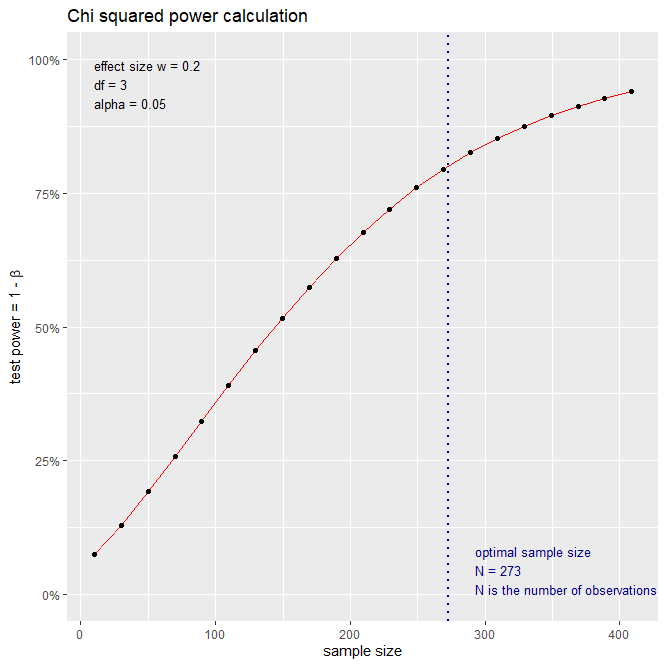


**Supplementary figure 2: HBsAg positivity as outcome - Covariates balancing checked by eQQ plots**


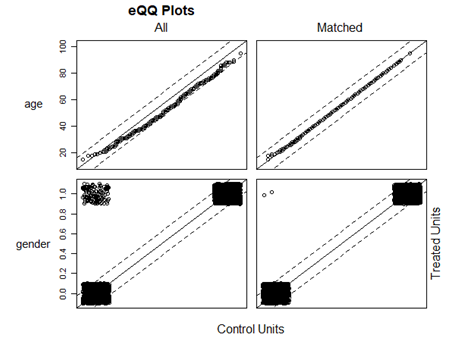


**Supplementary figure 3: HBsAg positivity as outcome - Covariates balancing checked by distribution of propensity scores**


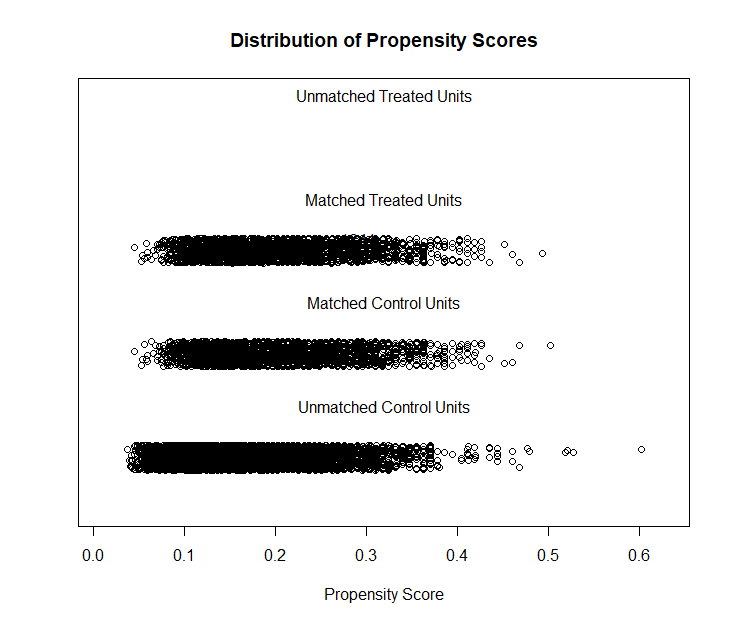

Supplement: Supplementary file 1 [file Data_Sheet_1.docx]
